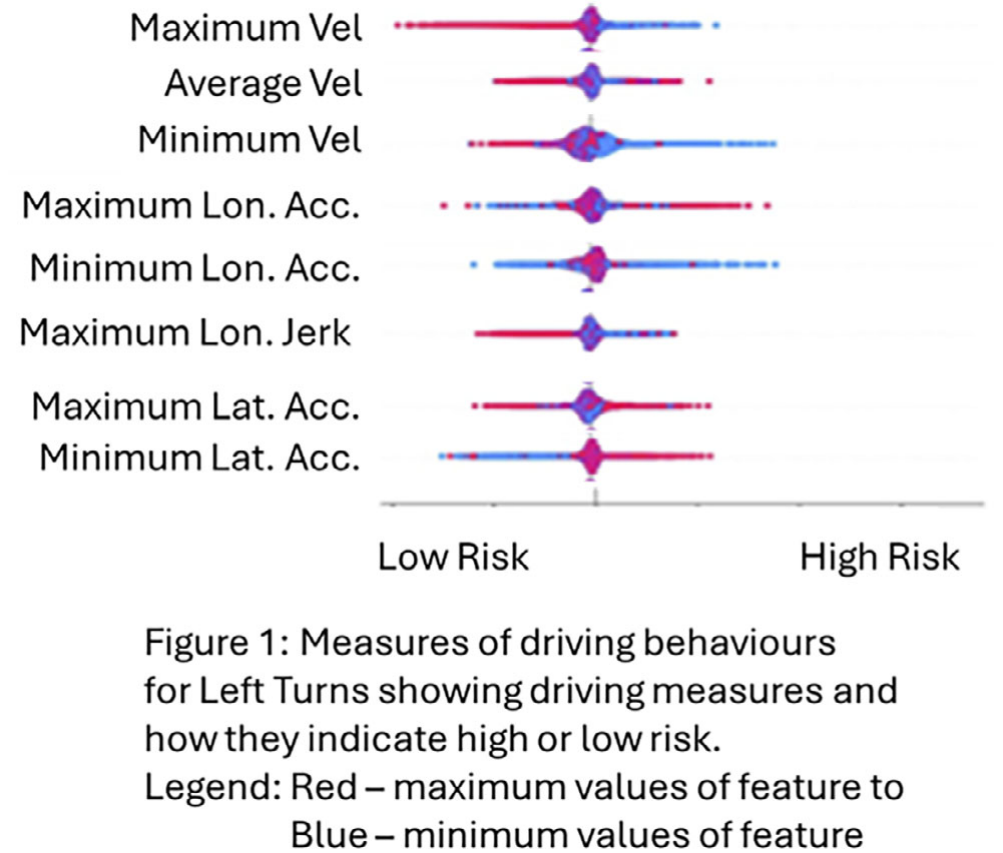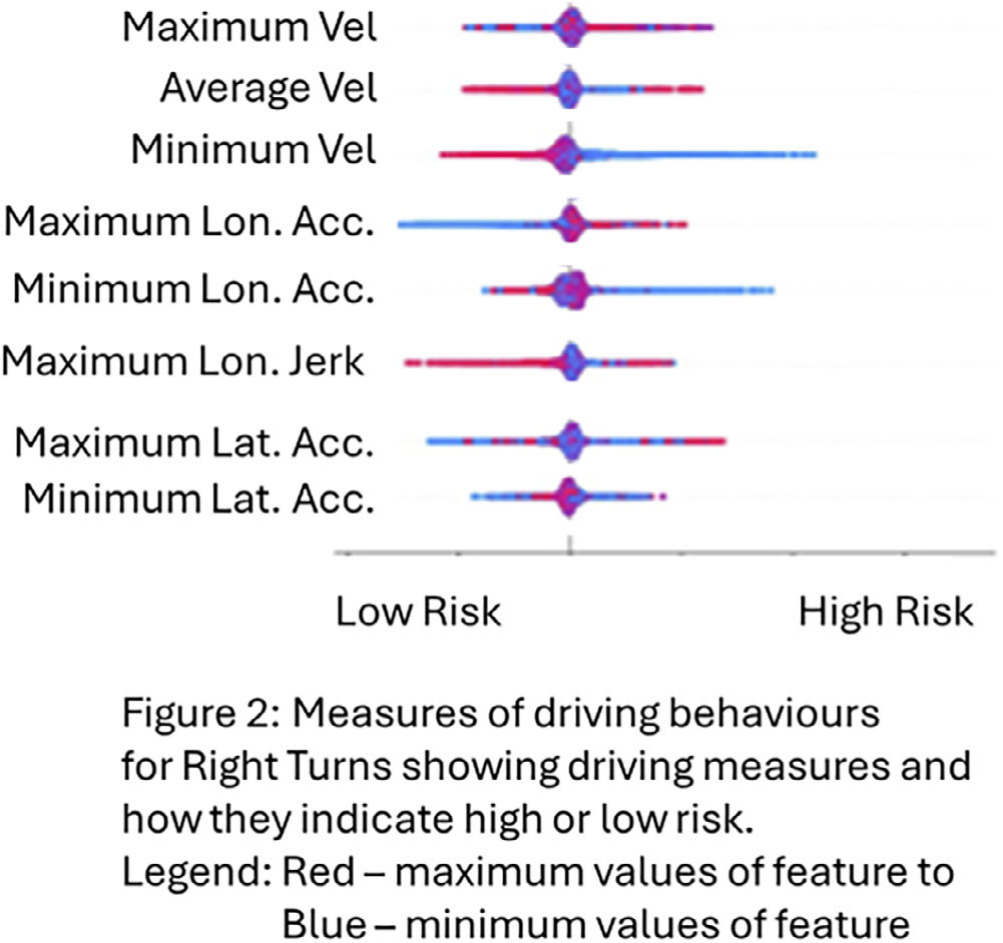# Identifying Mature Driver Risk from Naturalistic Driving

**DOI:** 10.1002/alz70858_106191

**Published:** 2025-12-26

**Authors:** Malak Saif El Nasr, Phil Masson, Bruce Wallace, Kathleen Van Benthem, Chris Herdman, Jocelyn Keillor, Rafik Goubran, Frank Knoefel, Shawn Marshall

**Affiliations:** ^1^ Carleton University, Ottawa, ON, Canada; ^2^ Bruyere Health Research Institute, Ottawa, ON, Canada; ^3^ AGE‐WELL NIH SAM3, Ottawa, ON, Canada; ^4^ National Research Council of Canada, Ottawa, ON, Canada; ^5^ University of Ottawa, Ottawa, ON, Canada; ^6^ Bruyere Continuing Care, Ottawa, ON, Canada

## Abstract

**Background:**

Driving is essential for independence while relying on physical and cognitive abilities. Many mature drivers struggle with decisions to adjust or stop driving. Physicians in Ontario, Canada are required to report patients they believe are no longer safe to drive. Understanding driving risk is challenging, with limited tools to analyze individualized driving. This work uses a database of mature driver in‐car data to investigate behaviors predictive of increased risk. The Candrive Risk Stratification tool (RST) predicts driving collision risk based on clinical measures. This project explores if naturalistic driving parameters correlate to the RST. This work provides knowledge to assist drivers in monitoring enabling proactive decisions about driving behaviour and driving retirement.

**Method:**

Candrive dataset analysis had ethics approval from the Bruyère Health and Carleton Research Ethics boards. This dataset includes up to seven years of in‐car sensor and annual health assessments for 250 drivers >70 years of age in Ottawa. This work focuses on telematics, including speed‐related measures, lateral/longitudinal acceleration, and jerk (change in acceleration), to determine if they are predictive of driving risk as indicated by RST scores. Key telematics scenarios are used for left and right turns, both known to challenge mature drivers.

**Result:**

Results are presented in Figures 1 (Left Turns) and 2 (Right Turns) in the form of Shapley plots that provide a visualization of a given measure's predictive ability to indicate driver risk. Maximum and minimum measures of velocity show a clear indication of risk, with drivers with lowest minimum speeds having higher risk while average velocity results are inconclusive as the red (high average velocity) and blue (low) are mixed with no distinction. Low values of maximum longitudinal acceleration and minimum lateral acceleration are also related to lower risk.

**Conclusion:**

The work demonstrates the potential for ongoing driving assessment to provide new insights for clinicians, drivers, and families related to risk derived from actual driving. Individualized knowledge about driving risk supports proactive measures, such as retraining, to mitigate declines or to prepare for driving retirement. Data from driving provides timely knowledge to the mature driver and offers significant advantages as compared to waiting for clinical assessments.